# The MID-PIWI module of Piwi proteins specifies nucleotide- and strand-biases of piRNAs

**DOI:** 10.1261/rna.044701.114

**Published:** 2014-06

**Authors:** Elisa Cora, Radha R. Pandey, Jordi Xiol, Josh Taylor, Ravi Sachidanandam, Andrew A. McCarthy, Ramesh S. Pillai

**Affiliations:** 1 European Molecular Biology Laboratory, Grenoble Outstation, 38042 Grenoble, France; 2 Unit for Virus Host-Cell Interactions, University of Grenoble Alpes-EMBL-CNRS, 38042 Grenoble, France; 3 Department of Oncological Sciences, Icahn School of Medicine at Mt. Sinai, New York, New York 10029, USA

**Keywords:** Piwi, piRNA, MID, U1-bias, strand-bias

## Abstract

Piwi-interacting RNAs (piRNAs) guide Piwi Argonautes to suppress transposon activity in animal gonads. Known piRNA populations are extremely complex, with millions of individual sequences present in a single organism. Despite this complexity, specific Piwi proteins incorporate piRNAs with distinct nucleotide- and transposon strand-biases (antisense or sense) of unknown origin. Here, we examined the contribution of structural domains in Piwi proteins toward defining these biases. We report the first crystal structure of the MID domain from a Piwi Argonaute and use docking experiments to show its ability to specify recognition of 5′ uridine (1U-bias) of piRNAs. Mutational analyses reveal the importance of 5′ end-recognition within the MID domain for piRNA biogenesis in vivo. Finally, domain-swapping experiments uncover an unexpected role for the MID-PIWI module of a Piwi protein in dictating the transposon strand-orientation of its bound piRNAs. Our work identifies structural features that allow distinguishing individual Piwi members during piRNA biogenesis.

## Abstract (precis)

This paper presents the crystal structure of the MID domain of a Piwi Argonaute protein. Docking experiments show that this domain specifies recognition of the 5′ uridine of piRNAs. Domain-swapping experiments reveal an unexpected role for the MID domain indicating strand orientation of its bound piRNA.

## INTRODUCTION

Argonaute proteins are expressed in most organisms ranging from bacteria to animals and plants (Filipowicz et al. 2008; Kawamata and Tomari 2010; Meister 2013). They are built to bind 19- to 30-nt small RNAs and facilitate their action on RNA targets. Argonautes can be classified into two major clades: AGO and PIWI. The AGO clade members are broadly detected in almost all organisms, including archaea and bacteria. They show ubiquitous expression and bind ∼21-nt microRNAs or small interfering RNAs (siRNAs) that identify targets by base-pairing to control gene expression. The PIWI clade, on the other hand, is restricted to animals and further confined to the gonads, often displaying strong sex-specific expression patterns. Together with their 24- to 30-nt Piwi-interacting RNAs (piRNAs), they control transposon activity and are essential for fertility in animals (Ghildiyal and Zamore 2009; Malone and Hannon 2009).

Biogenesis of piRNAs is only beginning to be understood, but analysis of factor requirements and precursor RNA features points to the process being mechanistically distinct from that of other small RNA classes. Single-stranded, often spliced, kilobases-long RNAs transcribed from discrete genomic loci called piRNA clusters are major sources for piRNAs (Brennecke et al. 2007; Li et al. 2013). These are broken down in an apparently random fashion by a mysterious primary processing pathway into tens of thousands of individual ∼30-nt RNAs. The end result is a tremendously heterogeneous population of primary piRNAs which are characterized by a preference for a 5′ uridine (1U-bias), the basis for which is unknown. The primary piRNAs identify transposon targets by base-pair complementarity, with extensive pairing resulting in slicing of the target by the Piwi endonuclease. In addition to silencing the target, this slicing event is harnessed to generate the 5′ end of new piRNAs via the secondary biogenesis pathway or the Ping-pong cycle, a process by which the target itself becomes a substrate for piRNA generation (Brennecke et al. 2007; Gunawardane et al. 2007). Since primary and secondary processing feed into distinct Piwi clade members, this results in piRNA pools having opposing strand orientations with respect to transposon sequences. How the system recognizes distinct Piwi members for sorting piRNAs based on sequence strand-orientation is presently unclear.

**FIGURE 1.**
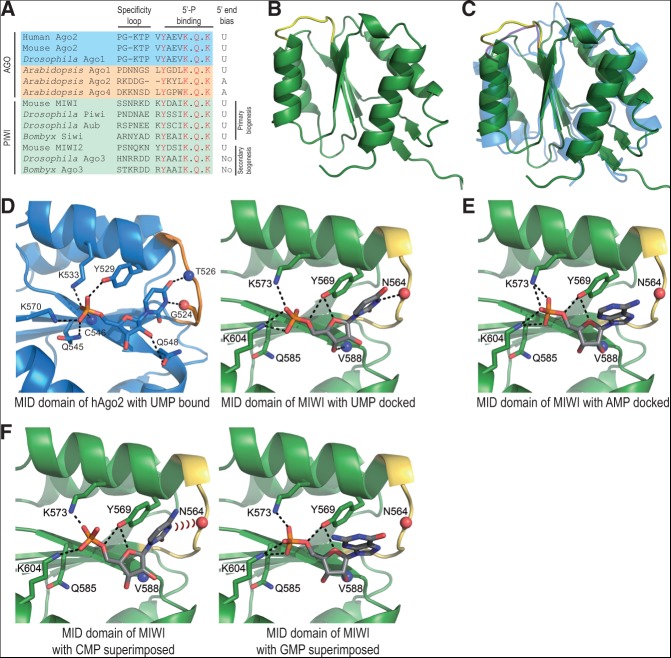
Crystal structure of the MID domain of the mammalian Piwi protein MIWI. (*A*) Sequence alignment shows key MID domain residues from indicated proteins; the specificity loop defining bias for UMP in hAgo2 and the residues (red) contacting 5′-monophosphate (5′-P) of the bound UMP in the hAgo2 structure (PDB: 3LUJ) are indicated. The 5′-P-binding residues are conserved in PIWI and AGO clade members of the Argonaute family. (*B*) Crystal structure of the MID domain of mouse Piwi protein MIWI (green) with the specificity loop highlighted (yellow). (*C*) Superimposition of the MIWI MID domain (green) and the MID of human Ago2 (hAgo2; blue) (PDB: 3LUC). Specificity loops of MIWI (yellow) and hAgo2 (magenta) are highlighted. (*D*) Comparison of the binding pockets of the MID domain of hAgo2 bound to UMP (blue) (PDB: 3LUJ) and the MID domain of MIWI with UMP docked (green). Specificity loops are shown in orange and yellow, respectively. Relevant atoms are indicated with blue spheres (nitrogen) and red sticks (oxygen), while dotted black lines indicate hydrogen bonds. (*E*) Docking solution of the MID domain of MIWI with AMP. The base nucleotide is too far to make a hydrogen bond with the specificity loop. (*F*) Representation of the superimposition of CMP on UMP and GMP on AMP when docked to the MID domain of MIWI.

Primary processing is credited with the origin of 1U-bias of piRNAs present in mouse MIWI and *Drosophila* proteins Aub and Piwi (Fig. 1A; Brennecke et al. 2007; Reuter et al. 2011). In contrast, secondary piRNAs like those present in *Drosophila* Ago3 do not show 1U-bias. One source of this bias could be the specificity of nuclease(s) generating the 5′ end of primary piRNAs. In this context, the Zucchini endonuclease acting in an unknown step during primary processing lacks any such specificity in vitro (Luteijn and Ketting 2013). Also complicating this simple correlation that 1U-bias is an obligate consequence of primary processing is the fact that mouse MIWI2 receives piRNAs via secondary processing, but still displays a preference for a 5′ U (Fig. 1A). Finally, given a chance to bind RNAs containing any of the 4 nt at the 5′ end, some Piwi proteins prefer to enrich only those carrying a 5′ U. This specificity is evident in the case of the two Piwi proteins expressed in the *Bombyx mori* (silkworm) ovary-derived BmN4 cells that participate in secondary processing via the Ping-pong cycle (Kawaoka et al. 2009). Within BmN4 cells, Siwi binds primary piRNAs with a 1U-bias, while Ago3 has secondary piRNAs lacking such a preference. Using a cell-free piRNA loading system, Tomari and colleagues (Kawaoka et al. 2011) found that this in vivo preference is maintained in vitro, with Siwi—but not Ago3—specifically enriching for 1U-containing synthetic RNAs. All these lead to an alternative possibility that the 1U-bias could be an inherent property of some Piwi proteins.

Argonautes are structurally organized into N-terminal, PAZ, middle (MID), and PIWI domains. Numerous crystal structures of archaeal, bacterial, and eukaryotic AGO proteins in complex with nucleic acids have indicated recognition of the 5′ monophosphorylated end within a conserved basic pocket formed by the MID domain, while the 3′ hydroxyl end is anchored in the PAZ domain (Parker and Barford 2006; Patel et al. 2006; Nakanishi et al. 2012; Schirle and MacRae 2012). The crystal structure of the isolated human Ago2 (hAgo2) MID domain first provided a structural basis for the enrichment of a 5′ uridine in microRNA sequences (Frank et al. 2010). Similarly, isolated MID domain structures of *Arabidopsis* AGO clade Argonaute members provided an explanation for enrichment of small RNAs with distinct 5′ nucleotide biases for a U, A, or C (Mi et al. 2008; Frank et al. 2012). This prompted us to examine the structural basis for 1U-bias of piRNAs through the analysis of the Piwi MID domain. Additionally, we also explored the role of Piwi structural domains in specifying sequence strand-biases of piRNAs.

## RESULTS AND DISCUSSION

Structural analysis of Piwi proteins is still in its infancy, with the PAZ domain being the only one characterized to date. The solution structure of mouse MIWI (Simon et al. 2011) and crystal structures of human Hiwi and Hili (Tian et al. 2011) reveal how the signature 2′-O-methyl modification at the 3′ termini of piRNAs is accommodated by the PAZ domain. To extend these studies, we crystallized the MID domain (S480-E616) of mouse MIWI, which binds primary piRNAs with a strong 1U-bias (∼90%) (Fig. 1A; Reuter et al. 2011). The native data set diffracted to a 2.3-Å resolution, but we were unable to solve the structure by molecular replacement using available AGO structures. Data collected from a crystal grown with selenomethionine allowed phasing by single-wavelength anomalous dispersion. The final structure was refined to 2.3 Å-resolution using the native data.

The overall structure of the MIWI MID domain reveals a Rossmann fold composed of alternating β-strands and α-helices that form a pocket capable of accommodating a single nucleotide (Rao and Rossmann 1973). The MIWI MID domain is characterized by a core four-stranded β-sheet flanked by four α-helices (Fig. 1B). It strongly resembles those found in prokaryotic and eukaryotic AGO proteins (Ma et al. 2005; Parker et al. 2005; Wang et al. 2009; Nakanishi et al. 2012; Schirle and MacRae 2012); nevertheless, the superimposition of MIWI and hAgo2 MID domains highlights differences in the orientation of several secondary structural elements and loops (Fig. 1C). The hAgo2 MID domain has been crystallized with soaked-in nucleoside monophosphates (NMPs) (Frank et al. 2010), but similar efforts with the MIWI MID domain failed. Consistently, physical interactions between MIWI MID and NMPs were not detected using a variety of methods including nuclear magnetic resonance (NMR). A likely explanation for this is the absence of the adjacent PIWI domain, which is known to contribute to the nucleotide-binding pocket in *Neurospora crassa* QDE-2 MID-PIWI and full-length eukaryotic Argonaute structures (Boland et al. 2011; Nakanishi et al. 2012; Schirle and MacRae 2012). In fact, *Neurospora crassa* QDE-2 MID-PIWI domain is required to bind RNA, while the MID domain alone is insufficient (Boland et al. 2011).

Therefore, we proceeded by positioning the ligands (NMPs) in the MIWI MID structure by docking analysis. In hAgo2, a proline-flanked rigid specificity loop (Fig. 1A) is shown to discriminate between the bases by making specific contacts only with uridine monophosphate (UMP) and adenosine monophosphate (AMP) (Frank et al. 2010). Comparison of the MIWI MID-UMP docking structure to that of hAgo2 MID-UMP shows that a group of conserved residues allow interaction with the 5′ phosphate group (MIWI residues: Y569, K573, Q585, and K604) and stacking of the base with a tyrosine (Y569 in MIWI) (Fig. 1D). The main difference occurs in the position of the specificity loop, which forces the base to change orientation. The specificity loop in MIWI MID lacks prolines (Fig. 1A) and is consequently more flexible, explaining the repositioning of the base. Docked AMP can also enter the binding pocket and makes a stacking interaction with Y569 but fails to engage N564 in the specificity loop via hydrogen bonding, as seen with docked UMP (Fig. 1E). Meanwhile, steric hindrance prevents GMP and CMP from binding (Fig. 1F). Thus, our docking studies suggest that the MIWI MID domain can optimally accommodate a 5′ uridine in the nucleotide-binding pocket, conferring an inherent ability to the protein in accumulating 1U-containing RNAs.

**FIGURE 2.**
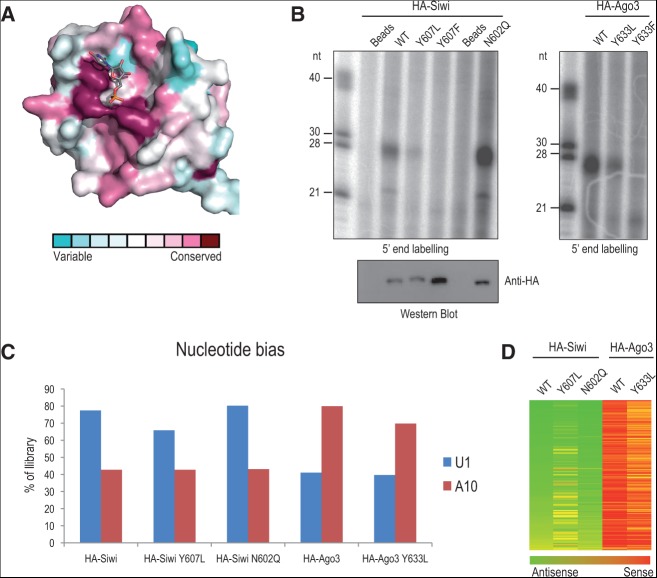
In vivo analyses of point mutations within the Piwi MID domain. (*A*) Surface conservation representation. Residues interacting with the 5′ phosphate of the small RNA are the most conserved within the nucleotide-binding pocket. (*B*) BmN4 cells were transiently transfected with indicated HA-tagged proteins and subjected to anti-HA immunoprecipitation. Small RNAs associated with HA-tagged protein complexes were revealed by 5′-end labeling. Single-stranded RNA markers are indicated in nucleotides (nt). Levels of precipitated proteins were verified by Western blotting. (*C*) Nucleotide preference for particular residues at positions 1 (U1-bias) and 10 (A10-bias) in indicated libraries. (*D*) Heat map showing strand-bias over 140 *Bombyx* transposon consensus sequences.

To further probe the MIWI MID crystal structure and obtain functional insights into piRNA biogenesis, we used the *Bombyx mori* BmN4 cell culture model. BmN4 expresses two Piwi proteins, Siwi (with 1U-bias) and Ago3 (without the bias), which we used for mutational analyses to probe the importance of the specificity loop and 5′ phosphate recognition within the conserved MID domain (Fig. 2A). First, we created variant *Bombyx* Piwi proteins, where we exchanged the entire loop between Siwi and Ago3 (loop-swap mutants). Unfortunately, these were not loaded with piRNAs in vivo. So, to avoid drastic changes to the protein structure, we focused on a single asparagine (N) in the specificity loop of Siwi for mutagenesis (Fig. 1A). Such an asparagine in the specificity loop of MIWI MID revealed the potential for hydrogen bonding with the base of UMP in our docking model (Fig. 1D). Furthermore, interaction with an asparagine within the specificity loop is shown to be crucial for specifying 1U-bias in the *Arabidopsis* AGO clade member AGO1 (Mi et al. 2008; Frank et al. 2012). To test its relevance, the specificity loop of *Bombyx* Siwi was mutated by converting the asparagine to a glutamine (N602Q). After expression in BmN4 cells, immunoprecipitation and 5′-end labeling revealed unaffected levels of bound small RNAs (Fig. 2B). Furthermore, deep sequencing revealed Siwi^N602Q^ to have 1U-bias identical to that found in wild-type HA-tagged Siwi protein (Fig. 2C). Alignment of these reads to transposon consensus sequences also confirmed the predominant antisense bias similar to that found in wild-type Siwi (Fig. 2D). These results are consistent with N564 in MIWI providing a backbone rather than a side-chain hydrogen bond. So, although we cannot experimentally demonstrate a direct role of the specificity loop in defining 1U-bias, our MIWI MID-UMP docking model suggests that the overall nucleotide-binding pocket of the MID domain in certain Piwi proteins may specify it.

Next, we mutated the conserved 5′ phosphate binding pocket within the Piwi MID domain (Fig. 2A). We substituted the stacking tyrosine (Y) in the two *Bombyx* Piwi proteins Siwi and Ago3 with either leucine (L) to abolish stacking or with phenylalanine (F) to maintain it, although both mutations are predicted to destroy hydrogen bonding with the 5′ phosphate of the bound small RNA (Fig. 2B). In both Siwi and Ago3, the two mutations drastically reduced piRNA association in vivo (Fig. 2B). We deep-sequenced the small amount of RNA recovered with Siwi^Y607L^ and Ago3^Y633L^ MID mutants. This revealed that, although reduced in small RNA levels, the nucleotide features (1U for Siwi and A10 for Ago3) (Fig. 2C) and transposon-strand orientations (Fig. 2D) of piRNAs loaded are unaffected. Our mutational analysis adds to previous work that analyzed the impact of Piwi MID mutations in vitro. Substitution of the tyrosine in *Bombyx* Siwi with glutamate (Y607E) or extension of the C-terminal end by a single alanine (A) residue (C-term +A), which presumably affects the C terminus's contribution to 5′ phosphate recognition within the MID domain (Boland et al. 2011; Nakanishi et al. 2012; Schirle and MacRae 2012), were found to drastically reduce small RNA binding in vitro (Kawaoka et al. 2011). Mutation within the MID domain in AGO clade members is also shown to drastically reduce small RNA binding in vivo (Boland et al. 2011; Rudel et al. 2011). Taken together, this confirms that 5′ phosphate recognition within the Piwi MID domain is essential for piRNA biogenesis in vivo and that the MID environment in some proteins may further specify the 5′-end nucleotide bias of the bound piRNA.

Another complex aspect within piRNA populations is the preferential incorporation of piRNAs with specific transposon strand-orientations into distinct Piwi proteins. This is exemplified by *Bombyx* Siwi and Ago3 expressed in BmN4 cells (Kawaoka et al. 2009). Siwi incorporates primary piRNAs with a 1U-bias (∼80%) that are predominantly antisense in orientation to the transposon consensus sequences. In contrast, Ago3 accepts secondary piRNAs that are generated by Siwi slicing on transposon transcripts, and as a consequence, Ago3-bound piRNAs are of sense orientation. Furthermore, Siwi and Ago3 piRNAs overlap across their 5′ ends by 10 nt, resulting in Ago3 piRNAs having a prominent A10-bias (∼80%), opposite to 1U of Siwi piRNAs. Ago3-bound piRNAs are then thought to participate in a feed-forward amplification loop that slices complementary cluster transcripts to provide more of the same Siwi piRNA that generated it. Thus, Siwi and Ago3 engage complementary transcripts and catalyze reciprocal cleavages to enhance piRNA levels via the Ping-pong cycle (Kawaoka et al. 2009), similar to the situation in *Drosophila* ovaries (Brennecke et al. 2007). Currently, it is not clear which structural features on the two proteins allow their discrimination by the piRNA biogenesis machinery.

**FIGURE 3.**
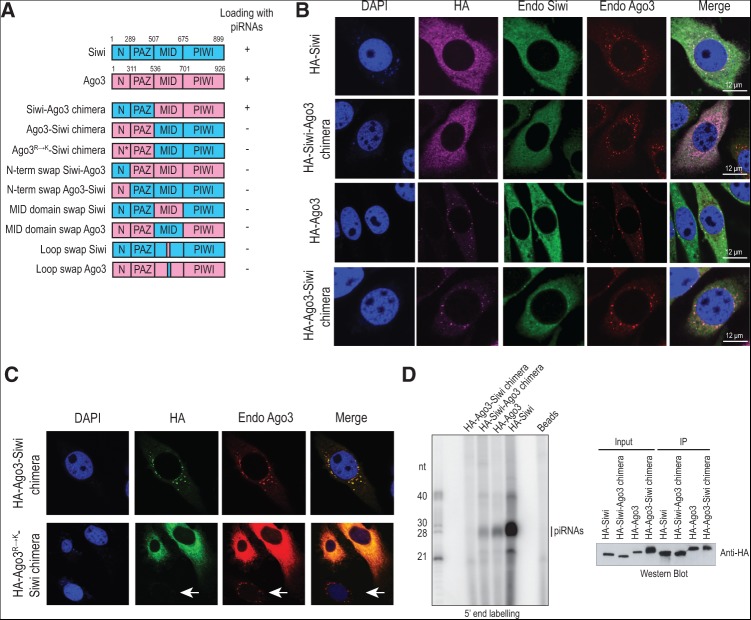
Subcellular localization of Siwi and Ago3 chimeric constructs. (*A*) Domain organization of Piwi proteins and their chimeric versions. Domain span (in number of amino acids) is indicated. The ability of the proteins to incorporate piRNAs in BmN4 cells is indicated. (*B*) Localization of HA-tagged Piwi proteins or their chimeric versions in BmN4 cells by immunofluorescence analysis (HA; magenta). Endogenous (Endo) Siwi (green) and Ago3 (red) were detected with specific polyclonal antibodies. Nuclei were stained with DAPI (blue). A merged image is shown. (*C*) Localization of the HA-Ago3-Siwi chimera and its mutated version harboring the R→K mutation on the N terminus of Ago3 by immunofluorescence analysis (HA; green). Endogenous Ago3 (red) was detected with a polyclonal antibody that recognizes the N terminus of the protein, resulting in a diffused staining in the cells that expressed the HA-Ago3^R→K^-Siwi chimera. Endogenous Ago3 was localized in the nuage in the untransfected cells (white arrow). (*D*) BmN4 cells were transiently transfected with indicated HA-tagged proteins and subjected to anti-HA immunoprecipitation. Small RNAs associated with HA-tagged protein complexes were revealed by 5′-end labeling. All proteins were expressed and immunoprecipitated as verified by Western blotting. Note that the HA-Ago3-Siwi chimera consistently lacked any bound RNAs.

To identify the domains that might distinguish the two Ping-pong Piwi partners, we prepared chimeric constructs where we swapped the N-PAZ domains of the proteins (Fig. 3A). For example, the Siwi-Ago3 chimera has N-PAZ of Siwi and MID-PIWI of Ago3. We first examined localization of the chimeric constructs, as the two Piwi proteins occupy different subcellular environments in BmN4 cells: Siwi is diffused in the cytoplasm, while Ago3 is enriched in peri-nuclear cytoplasmic granules called the nuage (Xiol et al. 2012). Immunofluorescence studies indicate that the chimeric constructs follow the localization pattern of the protein contributing the N-PAZ domain, with HA-Siwi-Ago3 chimera being cytosolic and HA-Ago3-Siwi chimera localized to the nuage (Fig. 3B). We believe that the N terminus alone is sufficient for guiding the nuage localization in the latter, as mutation of all N-terminal arginines (R) to lysines (K) in the chimera resulted in the HA-Ago3^R→K^-Siwi chimera losing its nuage accumulation and becoming diffused in the cytoplasm (Fig. 3C). We previously reported that a similar mutation in the context of Ago3 resulted in redistribution of the protein from the nuage to the wider cytoplasm, but this did not have any impact on the sequence profile of piRNAs in Ago3^R→K^ (Xiol et al. 2012). So, to test piRNA association with the chimeras, HA-tagged proteins were immunoprecipitated and bound small RNAs examined by 5′-end labeling (Fig. 3D). Repeatedly, only the Siwi-Ago3 chimera showed the presence of small RNAs. We prepared several additional mutants with single domain swaps (Fig. 3A) but did not manage to get Ago3-Siwi chimeric constructs that bound RNAs. We believe that structural incompatibilities of the fused domains might have resulted in proteins that are misfolded and nonfunctional.

**FIGURE 4.**
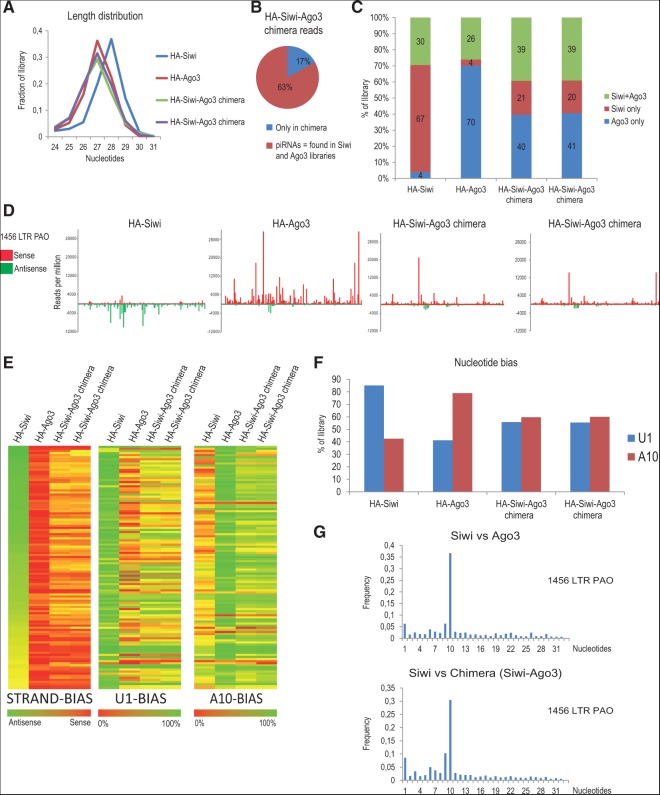
The MID-PIWI module influences transposon-strand bias of piRNAs. (*A*) Read-length distribution in the indicated libraries. (*B*) Classification of HA-Siwi-Ago3 chimera reads. Sequences present in HA-Siwi or HA-Ago3 libraries are referred as piRNAs, while the remaining sequences are indicated as “only in chimera.” (*C*) Sorting of piRNAs as Siwi-specific or Ago3-specific. A read is assigned to a particular Piwi protein if it is enriched by fivefold in that protein compared to the other. Where not possible, these are referred to as “Siwi+Ago3.” (*D*) Distribution of reads from indicated libraries over the 1456 LTR PAO *Bombyx* transposon consensus sequence. Sense (red) and antisense (green) piRNAs sort into distinct Ping-pong partners. (*E*) Heat map showing strand-bias, U1-bias, and A10-bias of reads over 118 *Bombyx* transposon consensus sequences. Note that the reads in Siwi-Ago3 chimera show a pattern similar to that of Ago3-bound piRNAs. (*F*) Nucleotide preference for particular residues at positions 1 (U1-bias) and 10 (A10-bias). (*G*) Correlation plot showing the distance between overlapping 5′ ends of reads mapping to the 1456 LTR PAO transposon consensus for the indicated libraries. The peak at position 10 indicates a 10-nt overlap that corresponds to the Ping-pong signature.

To examine the small RNAs associated with the HA-Siwi-Ago3 chimera, we prepared three independent deep-sequencing libraries (data from only two libraries are presented in Fig. 4). Sequences present in the HA-Siwi-Ago3 chimera libraries had a peak size of 27 nt, very similar to Ago3 piRNAs and different from those bound by Siwi (28 nt) (Fig. 4A). To precisely identify the reads, we compared the sequences present in the chimera to those already reported in Siwi and Ago3 libraries. Approximately 63% of the chimera reads can be found in Siwi and Ago3 complexes (Fig. 4B), but given the nonsaturating sequencing conditions, this number could be higher. Many of the reads in Siwi and Ago3 are shared between them, as an abundant read in one library can be found at least as a singleton in the other. So, we sorted reads based on an enrichment value (5×) to classify the reads as those found mainly in Siwi (Siwi-only) or Ago3 (Ago3-only). Those that could not be specifically assigned were grouped as Siwi+Ago3 reads. Based on such filtering, a substantial portion (∼40% Ago3-only) of the chimera reads can be attributed as being enriched in Ago3 (Fig. 4C). This is strikingly evident when reads are mapped onto the consensus sequence for the *Bombyx* transposon 1456 LTR Pao (Fig. 4D). Similar to Ago3, the chimera reads map to the sense strand of transposons, while Siwi reads derive from the antisense strand. Furthermore, this opposing polarity is maintained when reads are mapped onto over 118 *Bombyx* transposon consensus sequences (Fig. 4E).

Reads in the chimera have an intermediate 1U bias (55%), compared to that of Siwi (85%) and Ago3 (40%) (Fig. 4F). This indicates that the MID domain of Ago3 present in the chimera is unable to support a prominent 1U-bias. Nevertheless, calculation of nucleotide biases (U1 and A10) over various individual consensus sequences revealed biases that were largely similar to that found in Ago3 reads (Fig. 4E). Given the higher than expected 1U-bias of chimera piRNAs, it is possible that the MID domain alone may not be sufficient to specify the 5′-end bias of piRNAs, and there might be additional contributions from the piRNA processing pathway either to maintain a strong 1U-bias (as in primary processing) or enforce a lack of it (as in secondary processing). Based on the existence of strong Ping-pong signatures (the 10-nt overlap between their 5′ ends) between reads from Siwi and the chimera, secondary biogenesis initiated by endogenous Siwi is the source of much of the chimera-bound piRNAs (Fig. 4G).

Our deep sequencing analyses indicate that the MID-PIWI module of Ago3 within the HA-Siwi-Ago3 chimera gives it an Ago3-like identity. This was an unexpected finding. Argonaute proteins are defined by the presence of the signature PAZ, MID, and PIWI modules, with the N-terminal sequences being the most diverged, and generally used as antigens for creation of specific antibodies (as used in this study for *Bombyx* Siwi and Ago3). Furthermore, the N termini of Piwi proteins are decorated with a variable number of arginine residues that are targets for symmetrical dimethylation by PRMT5 (Heo and Kim 2009; Kirino et al. 2009). These marks are recognized by the Tudor domain in Tudor domain-containing proteins, many of which are confirmed piRNA biogenesis factors. Thus, the N termini of Piwi proteins were considered to be key features in distinguishing them during piRNA biogenesis. Our data described above now implicate the MID-PIWI module of Ago3 as a landmark to distinguish it from Siwi in the BmN4 cellular environment. It remains to be seen whether this is also true for Siwi in BmN4 cells and for other Piwi proteins in other systems.

Biogenesis of piRNAs begins in the nucleus with precursor transcription from piRNA clusters, but most biogenesis factors are localized to the cytoplasm, in peri-nuclear granules called nuages. How the nuclear history of a cluster transcript is transmitted to the cytoplasm is not known. One possibility is that specific RNA-binding proteins might tag cluster-originating transcripts to convey this information, distinguishing them from other abundant cellular mRNAs. Once in the cytoplasm, these chaperones, together with the primary piRNA biogenesis machinery, might probe the MID-PIWI module of individual Piwi proteins to deliver the precursors to the correct Piwi to mature them as primary piRNAs. Indeed, the secondary piRNA-accepting *Drosophila* Ago3 fails to get loaded when introduced into an environment that operates only the primary pathway, pointing to inherent differences within Piwi proteins (Olivieri et al. 2012). A similar mechanism to verify protein identity might operate in the secondary biogenesis pathway to deliver slicer cleavage products that mature as secondary piRNAs. There is precedence for the PIWI module of an AGO clade Argonaute to play a role in mediating interaction with Ago-interacting proteins (Till et al. 2007; Filipowicz et al. 2008). This is further illustrated by structural analysis of hAgo2, which revealed the presence of hydrophobic pockets in the PIWI module that can accommodate tryptophan (W) residues present in GW182 to mediate association between them (Schirle and MacRae 2012). Our studies show that the structural domains of Piwi proteins actively shape the piRNA population contained in them.

## MATERIALS AND METHODS

### DNA constructs

The MIWI MID domain (S480–E616) was cloned into pProExHtb vector (Invitrogen). HA-tagged *Bombyx* Siwi and Ago3 constructs are described (Xiol et al. 2012). Point mutations were introduced into the MID domains by an overlap PCR strategy. Chimeric Piwi protein constructs were created by swapping the N-PAZ domains: Siwi-Ago3 (Siwi [1–507] + Ago3 [537–926]) and Ago3-Siwi (Ago3 [1–536] + Siwi [508–899]). Additional swap mutants (see Fig. 3A) that were made are as follows: N-terminal swaps (Siwi [1–289] + Ago3 [312–926]) and (Ago3 [1–311] + Siwi [290–899]); MID domain swaps (Siwi [1–507 and 675–899] with Ago3 MID domain [537–701]) and (Ago3 [1–536 and 702–926] with Siwi MID domain [508–675]), and loop swaps (Siwi [1–599 and 606–899] with Ago3 loop [626–631]) and (Ago3 [1–625 and 632–926] with Siwi loop [600–605]). Arginines present in the N terminus of Ago3 within the Ago3-Siwi chimera were mutated to lysines as previously reported (Xiol et al. 2012).

### Expression, purification, crystallization, and data collection

The His_6_-tagged MIWI MID domain was expressed in *Escherichia coli* BL21 Rosetta cells. Soluble protein was purified by a Ni^2+^-affinity chromatography using chelating sepharose beads (GE Healthcare). Tag removal was performed by proteolytic digestion using Tobacco Etch Virus (TEV) protease and a subsequent Ni^2+^-affinity step. The protein was further purified by size-exclusion chromatography in 25 mM Tris-HCl, pH 8.0, 500 mM NaCl, 1 mM dithiothreitol (DTT), performed on a Superdex 75 10/300 GL column (GE Healthcare). The selenomethionine derivative (SeMet) of the MIWI MID domain (S480–E616) was expressed in *E. coli* B834 (DE3) cells grown in minimal M9 medium supplemented with 50 mg/mL L-SeMet (Sigma) and induced with 1 mM IPTG (EMBL Protein Expression and Purification Core facility). Purification was the same as the wild-type protein.

Crystals of native and SeMet-substituted MIWI MID domain were grown in sitting or hanging drops at 4°C from solutions composed of 100 mM Tris-HCl, pH 8.5, 100–200 mM MgCl_2_, and 26%–32% polyethylene glycol 4000. Crystals appeared after 3 d and were flash-frozen at 100K after transferring them to identical crystallization conditions containing 10% glycerol. The crystals were orthorhombic, space group P2_1_2_1_2_1_, contained four molecules in the asymmetric unit, and the best one diffracted to 2.3 Å. A highly redundant 2.8-Å SeMet anomalous data set was collected at the peak of the SeMet signal, as measured by X-ray fluorescence for experimental phasing. All X-ray data were collected on beamline ID14-4 (McCarthy et al. 2009) at the European Synchrotron Radiation Facility (ESRF), with integration and scaling carried out with the XDS suite (Kabsch 2010).

### Structure determination, refinement, and docking calculations

Auto-Rickshaw (Panjikar et al. 2005) was used to solve the structure. In summary, twenty SeMet sites were located on the basis of their anomalous differences using SHELXD (Schneider and Sheldrick 2002). These sites were refined, and experimental phases to 2.8 Å were calculated using the single anomalous dispersion (SAD) procedure in SHARP (de La Fortelle and Bricogne 1997). These phases were further improved by density modification and NCS averaging in DM, followed by model building with wARP (Morris et al. 2004). The initial model produced was positioned in the native data set with Phaser (McCoy et al. 2007). All subsequent refinement cycles were performed using REFMAC (Murshudov et al. 1997) with NCS restraints and a randomly chosen subset of 5% of reflections for the calculation of the free R-factor. Model building was carried out with Coot (Emsley and Cowtan 2004), and the stereochemical quality of the protein molecules was validated with Molprobity (Davis et al. 2007). All the crystallographic information is summarized in Table 1.

**TABLE 1.**
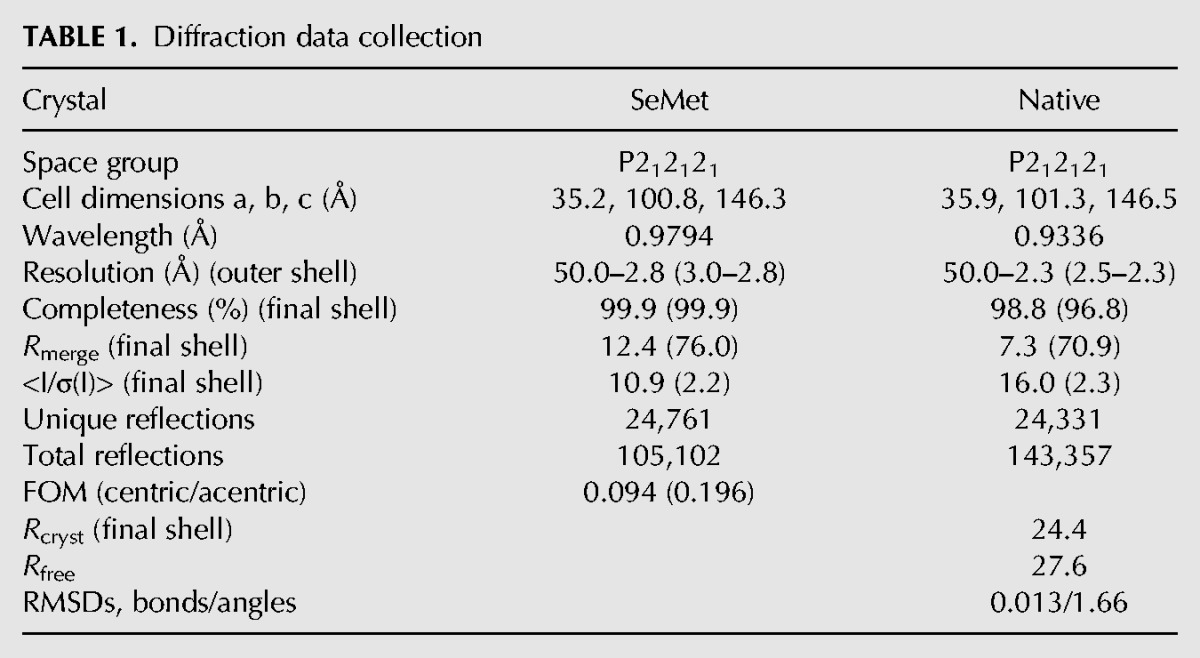
Diffraction data collection

AutoDock Tools (Morris et al. 2009) was used to prepare the ligand (AMP, CMP, GMP, and UMP) and receptor (MIWI MID domain) PDBQT files to include charges and hydrogen atoms. AutoDock Vina (Trott and Olson 2010) was then used for docking the ligands into a search box (30 × 30 × 30 Å^3^) centered on Y569. Surface conservation representation was based on sequence alignment of MID domains from the following: mouse Piwi proteins (MIWI, MILI, MIWI2) and *Bombyx* Piwi proteins (Siwi and Ago3), and human Ago2.

### *Bombyx* cell culture, immunoprecipitations, and Western blotting

The use of *Bombyx mori* ovarian cell line BmN4 for piRNA studies is described (Kawaoka et al. 2009). BmN4 cells were cultured at 27°C in IPL-41 medium (Life Technologies) supplemented with 10% fetal bovine serum (Life Technologies) and 2% penicillin:streptomycin (Life Technologies). Cell transfection was performed with 500 ng of expression plasmid for immunofluorscence (IF) assays (in 12-well plates) and with 2 µg for immunoprecipitation (IP) experiments (in 6-cm dishes), mixed with 2 and 5 µL Fugene HD reagent (Roche), respectively.

BmN4 cells were lysed in buffer (10% glycerol, 50 mM Tris-HCl, pH 8.0, 150 mM NaCl, 5 mM MgCl_2_, 5 mM DTT, 1× Complete EDTA-free protease inhibitor tablets (Roche) 0.5% Triton X-100, 50 μg mL^−1^ tRNA, and 1× vanadyl ribonucleoside complex [Sigma]). Immunoprecipitation of HA-tagged wild-type and mutant Piwi proteins was performed with HA-affinity beads (Roche). After multiple washes in IP buffer (10 mM Tris-HCl, pH 8.0, 150 mM NaCl, 1 mM MgCl_2_, 0.01% NP-40), immunoprecipitated samples were processed for Western blotting and extraction of associated RNAs. Bound RNAs were revealed by 5′-end labeling and 15% urea-PAGE. Western analyses were performed with mouse anti-HA antibody (kind gift of Marc Bühler) and anti-actin (Santa Cruz), both at a 1:200 dilution.

### piRNA library construction

Nucleic acids were isolated from immunoprecipitated HA-tagged protein complexes and resolved by 15% urea-PAGE. Bands corresponding to piRNAs were excised from the gel and extracted with 400 µL of 0.3 M NaCl solution at 25°C overnight. After purification by phenol-chloroform extraction, deep sequencing libraries were prepared using NEBNext Multiplex Small RNA Library Prep Set for Illumina (Cat. No. E7300) according to the manufacturer's instructions. Libraries were sequenced on an Illumina HiSeq platform (EMBL Heidelberg Gene Core facility). Small RNA analyses were as previously reported (Xiol et al. 2012).

### Immunofluorescence assay

BmN4 cells were grown on cover glasses and fixed with a 4% paraforamaldehyde solution (Sigma). Endogenous and HA-tagged Piwi proteins were detected using primary antibodies at a 1:200 dilution, while secondary antibodies coupled to Alexa 488, 594, and 647 (Invitrogen) were used for visualization at a 1:250 dilution. Antibodies used were the following: anti-Ago3 and anti-Siwi polyclonal antibodies (Xiol et al. 2012), and mouse anti-HA (gift of Marc Bühler). Nuclei were stained with DAPI (Sigma) by incubation for 30 min. Images were collected with a Leica TCD SP2 AOBS inverted microscope.

## DATA DEPOSITION

The crystallographic coordinates for the MIWI MID domain are deposited with the Protein Data Bank (PDB) under the accession code 4P1Z. Deep-sequencing data sets used in this study are deposited with Gene Expression Omnibus (GEO) under the accession number GSE55451.
